# Pathobiology of Second-Generation Antihistamines Related to Sleep in Urticaria Patients

**DOI:** 10.3390/biology11030433

**Published:** 2022-03-11

**Authors:** Caroline Mann, Joanna Wegner, Hans-Günter Weeß, Petra Staubach

**Affiliations:** 1 Department of Dermatology, Johannes Gutenberg University Mainz, 55131 Mainz, Germany; joanna.wegner@unimedizin-mainz.de (J.W.); petra.staubach@unimedizin-mainz.de (P.S.); 2 Division of Sleep Medicine, Center for Psychiatry, Psychosomatic and Psychotherapy, Pfalzklinikum Klingenmünster, 76889 Klingenmünster, Germany; guenter.weess@pfalzklinikum.de

**Keywords:** sleep, urticaria, antihistamines, quality of life

## Abstract

**Simple Summary:**

Sleep is a restorative state that is crucial for all human beings. Sleep is important for many biological processes in the body and has a huge impact on quality of life. According to previous studies, we know that patients with hives report sleep impairments. However, there are no data objectifying the sleep pattern. Guideline-based therapy for hives includes second-generation antihistamines of up to fourfold dosage. It is known that first-generation antihistamines lead to changes in sleep pattern and increased daytime sleepiness. However, the effect of second-generation antihistamines on sleep is not known. This pilot study was conducted to better understand the pathobiology of sleep in patients suffering from hives, who are medicated with high-dosed second-generation antihistamines. As healthy sleep in many dermatologic patients is still an unmet need, it is of utmost importance to raise awareness and eventually include sleep improvement in the therapy of urticaria patients.

**Abstract:**

Background: Standard treatment options for urticaria are second-generation antihistamines; however, their effect on sleep is uncertain. This study measures the influence of different antihistamines on the biologic sleep pattern of urticaria patients and the relevance of sleep in urticaria patients. Methods: Ten patients with chronic spontaneous urticaria (CSU) and uncontrolled symptoms under a single dose of second-generation antihistamines were included. Two nights were monitored: the first night after 5 days on single dosage and the second night after 5 days on fourfold dosage. Patient-rated questionnaires were used and sleep was monitored using polygraphy. Results: The patients’ rated daytime sleepiness decreased (*p* = 0.0319), as did their insomnia severity (*p* = 0.0349). The urticaria control (UCT) improved (*p* = 0.0007), as did the quality of life (*p* < 0.0001). There was no significant change of nightly pruritus (*p* = 0.1173), but there was an improvement of daytime pruritus (*p* = 0.0120). A significant increase in rapid eye movement (REM) sleep was seen (*p* = 0.0002) (from a mean of 3.9% to 14.3%). The deep sleep state (N3) also improved (8.7% to 12.3%) (*p* = 0.1172). Conclusion: This study has demonstrated an improvement of the sleep pattern in CSU patients under up-dosed second-generation antihistamines, without increased daytime sleepiness, alongside an improvement of urticaria symptoms and quality of life.

## 1. Introduction

Chronic spontaneous urticaria (CSU) is a common inflammatory dermatological disease with a high burden of disease. Symptoms include hives, pruritus and/or angioedema, resulting in a decrease in quality of life [1].

In previous studies, among others, we were able to show that sleep disturbances in patients with urticaria and atopic dermatitis is an underestimated burden [2,3,4,5]. However, there is great need for any data that can objectify sleep patterns in patients with chronic inflammatory skin diseases and pruritus, such as chronic urticaria. It is known that impaired sleep carries the risk of vascular events, i.e., stroke, coronary artery disease and myocardial infarction, and is also associated with obesity, impotence, and depression [6,7]. To what extent skin diseases have an influence on the sleep quality or vice versa is a matter of debate.

Sleep cycles can be measured with electroencephalography (EEG). It is part of polygraphy, a diagnostic tool that records multiple parameters during sleep [8]. The sleep architecture is characterized by the following stages [9]: non-rapid eye movement (NREM) sleep (which is defined by stages N1, N2 and N3) and rapid eye movement (REM) sleep. The waking stage is defined by high frequency (EEG waves 40–339 Hz), whereas light sleep (N1 and N2) is characterized by theta waves (4–8 Hz). Slow-wave sleep (N3) is characterized by low-frequency waves (0.5–4 Hz) and was found to be important for cognitive performance and memory consolidation [10]. REM sleep is characterized by predominant theta 6–9 Hz and gamma waves (30–300 Hz), with the disappearance of muscle tone and the occurrence of REM muscle twitches [9,11]. REM sleep is also known as the dream state and is crucial for memory consolidation and processing of sensory impressions [11,12]. The average sleep cycle begins with the NREM stage N1, constituting 2–5% of the whole sleep cycle, followed by N2, which constitutes about 45–55%, N3 (SWS), with 10–15%, and then REM, with 20–25% [8,11]. First-generation antihistamines are known to have a sedating effect, as a result of passing the brain–blood barrier and their anticholinergic side effects due to poor H1 receptor selectivity. Yanai et al. claim that the blocking of the H1 receptor by antihistamines is crucial for their sedative effect [13]. There are studies on first-generation antihistamines that show their use is associated with a decrease in REM sleep and REM-sleep latency [14,15]. Furthermore, there was an increase in daytime sleepiness, divided attention and vigilance [13,16]. Second-generation antihistamines have been developed to reduce these side effects [17]. The less sedating effect of second-generation antihistamines is explained by a lower H1-receptor occupancy [13] and a lower concentration in the central nervous system, following an active efflux through a pump in the blood–brain barrier [18]. There are reports based on patient-rated questionnaires [19,20] that show that even second-generation antihistamines, such as rupatadine and cetirizine, especially if up-dosed, lead to daytime sleepiness nonetheless. Second-generation antihistamines, up to fourfold dosage, are the first-line therapy for urticaria [21]. However, there is a lack of objective data showing the influence on the sleep quality under fourfold dosage of second-generation antihistamines in patients with chronic spontaneous urticaria.

## 2. Materials and Methods

The study protocol was approved by the ethics committee of the state of Rhineland-Palatinate, Germany. 

In our pilot study, 10 patients with CSU and uncontrolled symptoms under single doses of different second-generation antihistamines were recruited from October to November, 2020 from the Department of Dermatology at the University Medical Center in Mainz. No comorbidities or intake of comedication were reported. Each patient’s previously taken and well-tolerated antihistamine was continued (rupatadine in 7 patients, loratadine in 1 patient, desloratadine in 1 patient and cetirizine in 1 patient).

The patients were asked to fill out the following patient-reported outcomes.

### 2.1. Subjective Patient-Related Outcomes

Epworth Sleepiness Scale (ESS): Assesses daytime sleepiness. The ESS is an 8-item questionnaire, asking the patient to rate the likelihood of falling asleep during daily activities with low degrees of stimulation. The score ranges from 0 to 24 (with a score from 6 to 10 showing a higher normal daytime sleepiness, and 11 to 12 a mild excessive daytime sleepiness) (minimal clinical important difference (MCID) of 2–3 points) [22].

The Insomnia Severity Index (ISI) is a validated 7-item questionnaire (scoring from 0 to 28) asking patients to rate their current quality of sleep in order to assess the extent of insomnia. Quality of sleep as well as troubles “falling asleep”, “staying asleep” or “waking up too early” are rated by the patient. A score ranging from 8 to 14 would indicate subthreshold insomnia. The German version was validated by Gerber et al. in 2016 [23].

The Dermatological Life Quality Index (DLQI) is a common 10-item questionnaire with a range from 0 to 30 that measures the impact of different kinds of skin diseases on the quality of life during the previous week. A score value ≥10 indicates a severe impaired quality of life [24] (MCID of 3.3 points).

The UCT is a score that evaluates disease activity (0–16, with a score value ≥12 indicating disease control and a MCID of 3 points) [25]. The UCT score asks for physical symptoms, quality of life, treatment efficacy and urticaria control [5,26].

To score the pruritus severity, a numeric rating scale from 0–10 (NRS) was used for both daytime and nighttime pruritus (1: 0–2.9  =  mild, 2: 3–6.9  =  moderate, 3: 7–8.9  =  severe, 4: 9–10  =  very severe pruritus) (MCID of 3 points) [27].

All questionnaires were completed for both nights.

### 2.2. Objective Diagnostic Outcomes

Sleep was monitored for two nights, using a polygraphic device (Homesleep^®^ by somnomedics), an American Academy of Sleep Medicine (AASM)-certified and criteria-conformant device. The device is able to register 11 signals (3 frontoparietal EEG, 2 EOG, EMG, snoring, light, activity, head position and electrode impedance) [28]. The first evaluation was registered while patients were on a single-dosage of antihistamines and the second on day 6, after 5 days of fourfold dosage. All polygraphic channels were sampled with high- and low-pass filters. To avoid bias, the analysis of the polygraphic recordings was independent and blindly evaluated.

To eliminate the so-called “first night effect”, which can occur when patients spend their first night in a sleep laboratory, which potentially leads to restless sleep, the sleep was recorded at home in their usual surroundings [29].

## 3. Statistics

All data were assessed for normal or non-normal distribution. Differences in disease scores were determined using a paired *t*-test. The level of significance was set at α = 0.05. The resulting *p*-values were considered nominally significant at *p* ≤ 0.05. Statistical analyses were calculated with GraphPad Prism version 6.

## 4. Results

In our pilot study, 10 patients (8 female, 2 male) with an average age of 42.7 (SD ± 13.1) years, and a mean body mass index (BMI) of 26.9 (SD ± 4.5) were included. Fifty percent of the patients were overweight, and fifty percent had normal weight. Mean disease duration was 2.4 years (Table 1). There were no comorbidities or co-medications registered.

**Figure 1:**
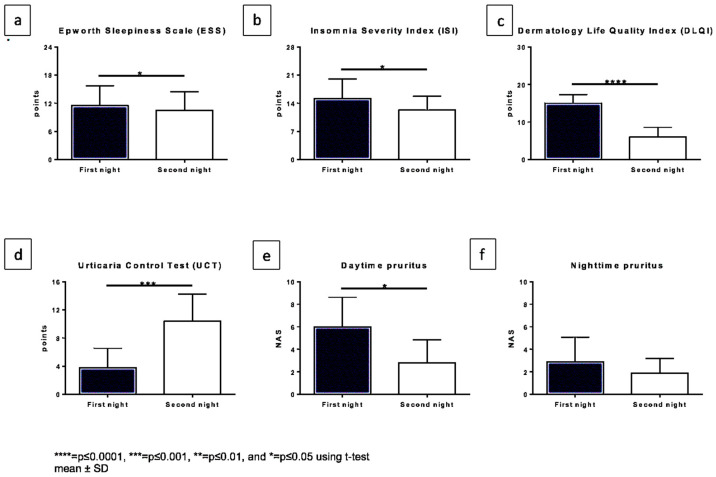
Changes in subjective patient-related outcomes comparing both nights. The x-axis displaying both nights and y-axis the score which was reached for: (**a**). Epworth sleepiness score, (**b**). Insomnia Severity Index (ISI), (**c**). Dermatology Life Quality Index (DLQI), (**d**). Urticaria Control Test (UCT) (a higher score in this case represents better symptom control), (**e**). Daytime pruritus NRS, (**f**). nighttime pruritus NRS.

Questionnaires: The ESS score for daytime sleepiness significantly decreased from 11.5 under a single dosage to 9.5 under a fourfold dosage of antihistamines (Figure 1a) (*p* = 0.0319).

The ISI score also showed a significant change from 15.2 to 12.3 (8–14 indicating subthreshold insomnia and 15–21 clinical insomnia (moderate severity)) (*p* = 0.0349) (Figure 1b).

The DLQI significantly improved (*p* < 0.0001) (Figure 1c) from a mean value of 15.0 to 6.0 under up-dosed antihistamines.

The UCT significantly increased from a mean of 3.8 to a mean of 10.4 (*p* = 0.0007) after 5 days (Figure 1d).

The pruritus during the night on the NRS changed from a mean value of 2.9 to 1.9 (*p* = 0.1173), whereas the pruritus during the daytime significantly improved from 6.0 under a single dosage to 2.8 under up-dosed antihistamines (*p* = 0.0120) (Figure 1e,f).

## 5. Polygraphy

**Figure 2:**
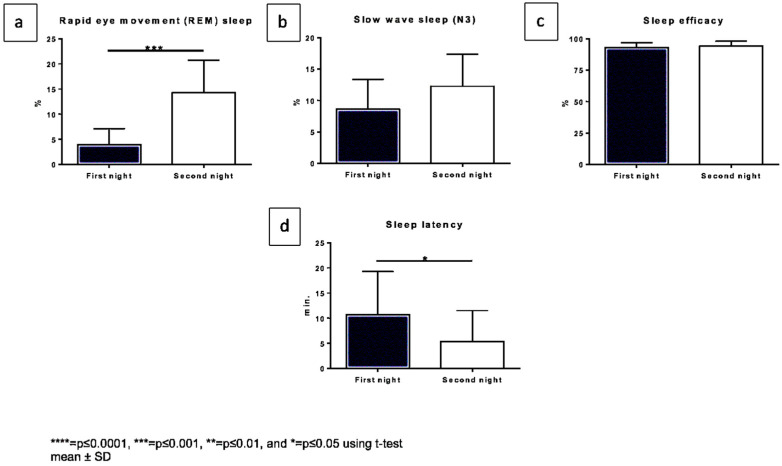
Changes in diagnostic polygraph outcomes comparing both nights. The x-axis displaying both nights, the y-axis: (**a**). REM sleep in % (**b**). Slow wave sleep (N3) in % (**c**). Sleep efficacy in %, (**d**). sleep latency in min.

Polygraphic results are displayed in Figure 2 and Table S1. The sleep duration showed a mean value of 7 h for all patients. We saw a significant (*p* = 0.0002) increase in REM sleep when comparing the first night under single dosage (mean of 3.9%) to the second night under fourfold dosage (14.3%) (Figure 2a). The deep sleep state (N3) also showed an increase (Figure 2b). There was only a slight difference in the sleep efficacy, (the first night with a mean of 93.1%, to the second night with a mean of 94.2%) (Figure 2c). The sleep latency significantly decreased (*p* = 0.0217) from a mean of 10.7 min on the first night to 5.4 min on the second night (Figure 2d).

## 6. Discussion

Improving sleep in patients with chronic inflammatory and itchy dermatological diseases, such as urticaria, is a highly worthwhile goal [30]. To best of our knowledge, there have been no data qualifying or quantifying sleep in chronic urticaria patients; however, sleep and quality of life are shown to be impaired.

Second-generation antihistamines can be up-dosed for the effective treatment of urticaria without increasing daytime sleepiness. Increasing their daily dosage up to fourfold, if necessary, is the recommended standard therapy for patients with chronic spontaneous urticaria, as per international guidelines [21]. Even at this elevated dosage, we did not notice any increase in daytime sleepiness in this pilot study, as documented with the ESS. In contrast, there was a reduction in the sleepiness score from 11.5 to 9.5, thus meeting the MCID criteria [31]. A reduction in daytime sleepiness might also hint at a more restful sleep.

It should be noted that both examinations were run in one week, and bigger differences could be expected after a longer intake of up-dosed antihistamines. The patients served as their own control group. Five of our ten patients had an elevated BMI; however, in only two of them was snoring recorded, which would make them candidates for obstructive sleep apnea, but this was not the topic of this study. Nonetheless, there was no difference in the sleep quality observed in these patients.

The ISI score showed a significant improvement in sleep quality but was still found to be elevated at 12.3 (8–14 subthreshold insomnia). This subjective rating was not reflected in the sleep architecture, as recorded with the EEG and EOG. However, this was also seen in other studies, who reported that subjective and objective quality of sleep often differ [32]. A potential placebo effect due to an increased number of tablets couldn’t be documented in our pilot study, as the ISI did not show a tremendous improvement [33].

In the literature, a first night effect is seen in patients undergoing polysomnograpy in a sleep laboratory, but this effect was not seen on sleep patterns in outpatient polygraphic examinations [34].

We saw that the quality of life (DLQI) improved significantly under up-dosed antihistamines (MCID: −3.3 points) [35]. The patients reporting a more restful sleep underline this. 

Up-dosed second-generation antihistamines, especially rupatadine, as it was taken by 7 of 10 patients, led to an increase in REM sleep, deep sleep and sleep efficacy. All those details signal a more restful sleep. No difference between the various antihistamines was observed in this small cohort. Despite an increase in REM sleep, there was no increase in nightmares reported by the patients. The fact that the sleep latency decreased is a positive sign, as “falling asleep” is one of the main obstacles when it comes to insomnia. On the one hand, this could be due to less urticaria disease activity. On the other hand, there are reports that even second-generation antihistamines, especially in higher doses [36], are able to cross the blood–brain barrier and interact with histamine neurons, which are essential for wakefulness [37].

Pruritus is often considered a main factor in impaired sleep in chronic inflammatory skin diseases [4,30,38,39]. Like in a previous study, the nightly pruritus reported by the patient in this study did not seem to be as severe as the perceived daytime pruritus [5]. The pruritus during daytime was rated as more severe than during the night and showed a significant improvement under up-dosed therapy. This is confirmed by looking at the first question of the DLQI, which asks how much the skin was burning, painful, sore or pruritic in the last days, on a scale from 3 (strong) to 0 (not present), and showed an improvement from a mean of 2.2 to a mean of 0.8. However, it also has to be taken into consideration that the minimal beneficial difference of the pruritus perceived by the patient is dependent on the baseline pruritus.

Thus, a missing statistically significant change in nighttime pruritus could still be of clinical relevance to the patient [27].

It remains speculative whether disease control led to secondary improvement of sleep quality or if it was due to the direct central effect of the up-dosed antihistamines. It would be interesting to see if patients with fully controlled urticaria symptoms under a single-antihistamine dose show the same sleep pattern, which would indicate the latter reason.

In this pilot study, 5 days of up-dosing with second-generation antihistamines in CSU patients led to an improvement of not only indirect but also direct sleep conditions, as documented by questionnaires and by polygraphic measurements. Control of pruritus was seen in some patients; however, as shown before, pruritus did not seem to be the subjectively decisive factor for every patient’s sleeping problem [5,40].

In some parts of the world, first-generation antihistamines are still used for the treatment of urticaria; however, this not only forms a potential risk for reduced REM sleep and daytime drowsiness, but also depression and other health problems, if overdosed [41]. Therefore, it is of great importance to advance research on second-generation antihistamines and further elaborate their benefits.

It is known that impaired sleep over time is highly associated with depression [7] and mental disorders, a frequent comorbidity in chronic urticaria patients [42]. Furthermore, quality of life is strongly associated with sleep quality [43]. Improving sleep in patients with urticaria by adequate treatment may have a broader therapeutic benefit and help to prevent further comorbidities. With this pilot study, we wanted to once more highlight the relevance of further studies to prove those findings [44]. Beyond that, investigations of sleep quality should be encouraged in new drug registration studies.

## 7. Conclusions

This study has demonstrated an improvement in sleep patterns in CSU patients under up-dosed second-generation antihistamines, without increased daytime sleepiness, alongside an improvement in urticaria symptoms and quality of life.

## Figures and Tables

**Table 1:** Descriptive characteristics (mean ± SD) of the studied group.

Item	
Sex, n	
total	10
women	8
men	2
Age, years, mean ± SD	42.7 ± 13.1
range	27–63
BMI, kg/m^2^, mean ± SD	26.9 ± 4.5
range	21.8–36.7
urticaria duration, years, mean ± SD	2.4 ± 1.7
range	0.5–4

## Data Availability

The datasets generated and analyzed during the current study are available from the corresponding author upon reasonable request.

## Supplementary Materials

The following supporting information can be downloaded at: https://www.mdpi.com/article/10.3390/biology11030433/s1, Table S1: Descriptive characteristics of the sleep and disease parameters comparing both nights.
