# Supplementary material for: Pathobiology of Second-Generation Antihistamines Related to Sleep in Urticaria Patients
Source: Biology (Basel). 2022 Mar 11;11(3):433. doi: 10.3390/biology11030433 (PMC8945773; doi:10.3390/biology11030433)
Supplement: Supplementary file 1 [file biology-11-00433-s001.zip › biology-1563292-supplementary.pdf]

Table S1: **Descriptive characteristics of the sleep and disease parameters comparing both nights**

Item	First night	Second night	p-value
**REM sleep in %**			
mean ± SD; range	3.9 ± 3.2; 0 – 9.2	14.3 ± 6.4; 8,4 – 26	0.0002
**Slow wave sleep (N3) in %**			
mean ± SD; range	8.7 ± 4.7; 0 – 15	12.3 ± 5.1; 4.4 -19.2	0.1172
**Sleep efficacy in %**			
mean ± SD; range	93.1 ± 3.7; 84.3 – 97.3	94.2 ± 3.9; 84.1 - 98.6	0.1202
**Sleep latency in min.**			
mean ± SD; range	10.7 ± 8.6; 1.5 – 31	5.4 ± 6.1; 0 - 20.4	0.0217
**Epworth Sleepiness Scale (ESS)**			
mean ± SD; range	11,5 ± 4.2; 5.0 – 18	10.5 ± 4.0; 4.0 –18	0.0319
**Insomnia Severity Index (ISI)**			
mean ± SD; range	15.2 ± 4.8; 6.0 – 23	12.3 ± 3.4; 6.0 - 18	0.0349
**Urticaria Control Test (UCT)**			
			0.0007
mean ± SD; range	3.8 ± 2.7; 0 – 8	10.4 ± 3.8; 5.0 – 16	
**Dermatology Life Quality Index(DLQI)**			
mean ± SD; range	15.0 ± 2.3; 13.0 – 21	6.0 ± 2.6; 0 – 10	< 0.0001
**Daytime pruritus on NAS**			
mean ± SD; range	6.0 ± 2.6; 2.0 – 10	2.8 ± 2.0; 0 – 6	0.0120
**Nighttime pruritus on NAS**			
mean ± SD; range	2.9 ± 2.2; 0 – 6	1.9 ± 1.3; 0 – 4	0.1173
